# Coexistence of Colorectal Cancer and Immunoglobulin G4-Related Disease in the Same Lesion: A Rare Case with Molecular Classification

**DOI:** 10.3390/diagnostics14020138

**Published:** 2024-01-08

**Authors:** Kiyong Na, So-Woon Kim

**Affiliations:** Department of Pathology, Kyung Hee University Hospital, Kyung Hee University College of Medicine, Seoul 05278, Republic of Korea; naky0430@khu.ac.kr

**Keywords:** colonic neoplasm, immunoglobulin G4-related disease, coexistence of cancer and IgG4-related disease

## Abstract

Immunoglobulin G4-related disease (IgG4-RD) is a novel fibroinflammatory disorder characterized by enlargement of the involved organs, elevated IgG4 levels, and abundant infiltration of IgG4-positive plasma cells. Indeed, primary colon cancers arising from IgG4-RD are rare. This case report describes a rare occurrence of simultaneous colorectal cancer and IgG4-RD in the same lesion in a 62-year-old male patient. The patient underwent a right hemicolectomy under the suspicion of primary colon cancer. The mass was grossly well-defined and yellowish tan, and the background colon was fibrotic. Microscopically, the tumor cells showed glandular differentiation characteristic of adenocarcinoma in a background of dense lymphoplasmacytic infiltration with fibrosis and obliterative phlebitis in the pericolic fat tissue. IgG4 immunohistochemical staining showed diffuse positivity in infiltrating plasma cells. The patient was administered adjuvant chemotherapy and prednisolone therapy. The patient’s serum IgG4 levels gradually decreased, and a follow-up positron emission tomography-computed tomography scan 1 year after surgery showed no evidence of local or distant recurrence of colorectal cancer. IgG4-RD occurring concurrently with primary colon adenocarcinoma has not been reported. Increased awareness of this rare coexistence can guide clinicians in navigating diagnostic complexities and selecting optimal therapeutic strategies.

Immunoglobulin G4-related disease (IgG4-RD) is a rare systemic fibroinflammatory disorder that can affect multiple organs and mimic malignancies [1]. Although the pathogenesis of IgG4-RD is not fully understood, it is considered an autoimmune or allergic disorder characterized by lymphoplasmacytic infiltration with IgG4 immunoreactivity, storiform fibrosis, and obliterative phlebitis [2]. The clinical manifestations of IgG4-RD vary depending on the involved organs and can include autoimmune pancreatitis, retroperitoneal fibrosis, salivary gland swelling, and lymphadenopathy [3,4].

Although a complete consensus has not yet been established, patients with IgG4-RD are typically considered to be at high risk of developing malignant diseases [5,6,7,8]. Although the association between IgG4-RD and malignancy is not well established, several studies have reported the coexistence of IgG4-RD and various cancers, including pancreatic, lung, and breast [9]. It has been suggested that chronic inflammation and tissue damage in IgG4-RD may predispose patients to developing malignancies. Furthermore, some cases of IgG4-RD may represent paraneoplastic syndrome or a reaction to a tumor antigen [10]. With recent associations between IgG4-RD and malignancies, this distinction is crucial, as treatment options and prognoses differ significantly [5,11,12,13,14,15].

This report presents a rare patient with a synchronous occurrence of carcinoma underlying an isolated colon mass in the context of IgG4-RD. Here, we discuss the patient’s clinical presentation, diagnostic evaluation (including molecular analysis), and management to facilitate a better understanding of this condition.

The patient was a 65-year-old male with no underlying medical condition who presented with a 4-month history of worsening abdominal pain, weight loss, and constipation. Physical examination results were unremarkable, except for mild abdominal tenderness in the left lower quadrant. Laboratory investigations revealed an elevated serum carcinoembryonic antigen level of 28 ng/mL (normal range, <5 ng/mL).

Colonoscopy revealed a circumferential irregular mass with luminal narrowing in the transverse colon (T-colon), and biopsy revealed adenocarcinoma (Figure 1A). Magnetic resonance imaging (MRI) revealed irregular concentric wall thickening with stenosis at the proximal T-colon near the hepatic flexure (approximately 6 cm in extent), which was likely T-colon cancer (cT4aN2Mx) (Figure 1B). Multifocal extramural venous invasion, pericolic nodular extension, and serosal thickening with omental infiltration were also observed, suggesting an advanced stage of colon cancer. To further evaluate this finding, the patient underwent positron emission tomography-computed tomography (PET-CT), which revealed hypermetabolic wall thickening (SUV_max_ = 10.9) in the T-colon with pericolic infiltration and subtle hypermetabolic pericolic lymph nodes (Figure 1C). No other organs were involved except for the colon.

The patient underwent right hemicolectomy. The mass was grossly well-defined and yellowish tan, and the background colon was fibrotic. Microscopically, the tumor cells showed glandular differentiation with atypical nucleus and mitosis, confirming the presence of moderately differentiated adenocarcinoma invading the muscularis propria and pericolic adipose tissue in the transverse colon (Figure 2A,B). Moreover, there was dense lymphoplasmacytic infiltrate, obliterative phlebitis, and storiform fibrosis adjacent to and below adenocarcinoma in the resected colon specimen (Figure 2C–E). IgG4 immunostaining showed increased IgG4-positive plasma cells with an IgG4/IgG ratio of 60% in the colon specimen, supporting the diagnosis of IgG4-RD (Figure 2F).

Next-generation sequencing was performed on hybridization-captured, adaptor ligation-based libraries using DNA extracted from 4 μm, formalin-fixed, paraffin-embedded sections from the present case. The NGS analyis revealed a somatic mutation in the *KRAS* gene at codon 12 (c.35G>A, p.G13D), which is a known driver mutation in colorectal cancer. Additionally, *APC* and *TP53* truncating mutations (c.3340C>T, p.R1114* and c.637C>T 17, p.R213*, respectively) were noted. No mutations were detected in *BRAF*, *NRAS*, or *PIK3CA*, and no copy number alterations were observed. Furthermore, no microsatellite instability or mismatch repair deficiency was detected, suggesting proficient mismatch repair status.

Given the coexistence of colorectal cancer and IgG4-RD, the patient was noted to have elevated serum IgG4 levels (302 mg/dL; normal range, <135 mg/dL) on laboratory testing. The patient was administered adjuvant chemotherapy with folinic acid, fluorouracil, and oxaliplatin (FOLFOX) regimen for colorectal cancer and prednisolone (30 mg daily) for IgG4-RD. During prednisolone therapy, the patient’s serum IgG4 levels gradually decreased (302 to 51 mg/dL), and a follow-up PET-CT scan 1 year after surgery showed no evidence of local or distant recurrence of colorectal cancer. The patient is currently being closely followed-up with using surveillance imaging and laboratory tests.

## Figures and Tables

**Figure 1:**
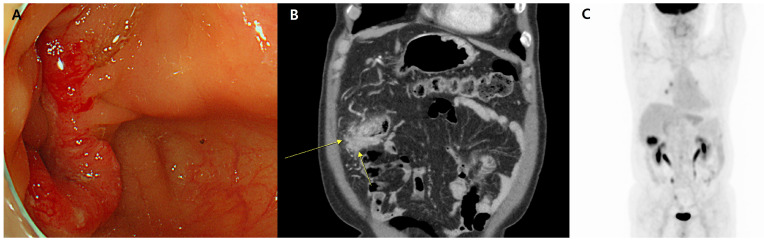
Endoscopic and radiologic images. (**A**) Endoscopic image revealing a prominent mass lesion in the transverse colon, characterized by mucosal irregularity and friability. (**B**) Axial T2-weighted magnetic resonance imaging (MRI) depicting the transverse colon that presents irregular concentric wall thickening with stenosis at the proximal transverse colon near the hepatic flexure, extending approximately 6 cm (arrows). These findings suggest transverse colon cancer (cT4aN2Mx) and demonstrate multifocal extramural venous invasions as well as pericolic nodular extensions, serosal thickening with enhancement, and omental infiltration. (**C**) Positron emission tomography-computed tomography (PET-CT), which revealed hypermetabolic wall thickening in the T-colon with pericolic infiltration and subtle hypermetabolic pericolic lymph nodes. No other organs were involved except for the colon.

**Figure 2:**
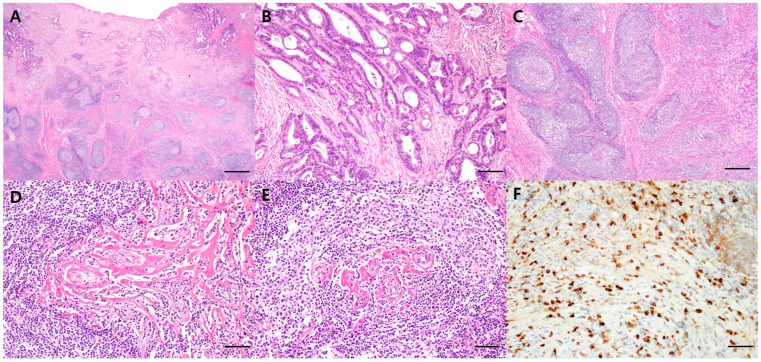
Representative hematoxylin and eosin (H&E) and immunohistochemical staining images of the specimen. (**A**) H&E staining showing infiltrating cancer (upper) associated with dense lymphoplasmacytic lesion and fibrosis (lower). (**B**) A well-formed glandular lesion with atypical cells is observed, consistent with adenocarcinoma. (**C**–**E**) In the lower part, dense lymphoplasmacytic infiltrate and storiform fibrosis are observed, along with obliterative phlebitis. (**F**) Immunohistochemical staining highlights an increased number of IgG4-positive cells (brown staining) in the colon specimen. These histopathological features are characteristic of IgG4-related disease, indicating immune system-related inflammation and fibrosis in the colon tissue (original magnification: (**A**), scan view; (**B**), 200×; (**C**), 100×; (**D**–**F**), 400×).

## Data Availability

The data presented in this study are available upon request from the corresponding author. The data are not publicly available because of privacy and ethical restrictions.

## References

[1] Deshpande V., Zen Y., Chan J.K. (2012). Consensus statement on the pathology of IgG4-related disease. Mod. Pathol..

[2] Umehara H., Okazaki K., Masaki Y. (2012). Comprehensive diagnostic criteria for IgG4-related disease (IgG4-RD). Mod. Rheumatol..

[3] Pieringer H., Parzer I., Wöhrer A., Reis P., Oppl B., Zwerina J. (2014). IgG4-related disease: An orphan disease with many faces. Orphanet J. Rare Dis..

[4] Kamisawa T., Zen Y., Pillai S., Stone J.H. (2015). IgG4-related disease. Lancet.

[5] Hirano K., Tada M., Sasahira N. (2014). Incidence of malignancies in patients with IgG4-related disease. Intern. Med..

[6] Shiokawa M., Kodama Y., Yoshimura K. (2013). Risk of cancer in patients with autoimmune pancreatitis. Am. J. Gastroenterol..

[7] Asano J., Watanabe T., Oguchi T. (2015). Association between immunoglobulin G4-related disease and malignancy within 12 years after diagnosis: An analysis after longterm follow-up. J. Rheumatol..

[8] Schneider A., Hirth M., Münch M. (2017). Risk of cancer in patients with autoimmune pancreatitis: A single-center experience from Germany. Digestion.

[9] Hamano H., Kawa S., Horiuchi A. (2001). High serum IgG4 concentrations in patients with sclerosing pancreatitis. N. Engl. J. Med..

[10] Fei Y., Shi J., Lin W. (2015). Intrathoracic involvements of immunoglobulin G4-related sclerosing disease. Medicine.

[11] Yamamoto M., Takahashi H., Tabeya T. (2012). Risk of malignancies in IgG4-related disease. Mod. Rheumatol..

[12] Yu T., Wu Y., Liu J., Zhuang Y., Jin X., Wang L. (2022). The risk of malignancy in patients with IgG4-related disease: A systematic review and meta-analysis. Arthritis Res. Ther..

[13] Tang H., Yang H., Zhang P., Wu D., Zhang S., Zhao J., Peng L., Chen H., Fei Y., Zhang X. (2020). Malignancy and IgG4-related disease: The incidence, related factors and prognosis from a prospective cohort study in China. Sci. Rep..

[14] Sodavarapu S., Ghotra G.S., Obad N., Goyal M., Gill A.S. (2019). IgG4-Related Diseases–Continues To Be a Cancer Mimicker. Cureus.

[15] Lemaitre S., Esquerda G.M., Guardiola A.C., Jordi T., Sanda N., González-Candial M. (2018). Colon cancer and IgG4-related disease with orbital inflammation and bilateral optic perineuritis: A case report. Medicine.

